# InfraRed Thermographic Measurements in Parkinson’s Disease Subjects: Preliminary Results

**DOI:** 10.3390/s25175243

**Published:** 2025-08-23

**Authors:** Antonio Cannuli, Fabrizio Freni, Antonino Quattrocchi, Carmen Terranova, Andrea Venuto, Roberto Montanini

**Affiliations:** 1 Department of Engineering, University of Messina, C.da di Dio, 98166 Messina, Italy; fabrizio.freni@unime.it (F.F.); antonino.quattrocchi@unime.it (A.Q.); andrea.venuto@unime.it (A.V.); roberto.montanini@unime.it (R.M.); 2 Department of Clinical and Experimental Medicine, University of Messina, Via Consolare Valeria, 1, 98125 Messina, Italy; carmen.terranova@gmail.com

**Keywords:** infrared thermography, Parkinson’s disease, autonomic dysfunction, thermoregulation

## Abstract

In this preliminary study, the thermoregulatory response in individuals diagnosed with Parkinson’s disease was investigated by infrared thermography. Parkinson’s disease is a complex neurodegenerative disorder primarily known for motor impairments, significantly reducing the quality of life of affected people. However, in most cases, such disease is accompanied or preceded by non-motor symptoms, including autonomic dysfunction. As in the case of neurovegetative dysautonomia, this dysfunction involves a malfunction of the autonomic nervous system, which also plays a key role in thermoregulation. In general, such conditions are not always easy to detect; a valid method could be represented by the vasomotor response of the skin to cold stimuli. In this context, infrared thermography can provide insights into the thermoregulatory patterns associated with autonomic dysfunction, representing a valuable tool for non-invasive assessment of Parkinson’s research. Early biomarkers of the disease can be obtained through changes in skin temperature, allowing for timely intervention and management. The study was conducted on a cohort of 16 subjects (8 patients with Parkinson’s disease and 8 healthy controls), who were monitored with infrared images captured from their hands, following a specific protocol established by a preliminary analysis. Experimental results revealed that thermography can detect focal points and regions exhibiting either hyper- or hypothermia across the skin surface and muscular regions. This capability allows for extracting and categorizing precise medical data, which could inform future research aimed at identifying early markers of the disease. However, as this is a preliminary observational study, no diagnostic claims are made, and further investigations on larger cohorts with controlled comorbidities are needed.

## 1. Introduction

Infrared thermography (IRT) is a non-invasive imaging technique that detects and visualizes the infrared radiation emitted by bodies with temperatures above absolute zero, translating it into thermal images, i.e., thermograms.

The temperature distribution obtained can reflect physiological or pathological processes occurring in superficial tissues. Since its first medical application in breast cancer screening in the 1950s [1], IRT has experienced significant technological evolution. Improvements in spatial resolution, thermal sensitivity, portability, and cost-effectiveness have made it increasingly suitable for biomedical applications.

Today, IRT is employed in several clinical and research contexts, including the study of vascular disorders, musculoskeletal and rheumatic conditions, dermatological diseases, fever screening, and neurological pathologies [2,3,4,5,6,7,8,9,10,11]. In particular, it enables the evaluation of skin temperature dynamics in real time, offering a sensitive window into autonomic nervous system (ANS) activity and dysfunction. When used in combination with standardized protocols and environmental controls, thermography allows objective quantification of thermoregulatory responses to external stimuli. Parkinson’s disease (PD) is a progressive neurological disorder that affects the central and peripheral nervous system, giving rise to bradykinesia, tremor, rigidity, and difficulty with balance and coordination. It is caused by a degeneration of dopaminergic neurons in substantia nigra, striatal projections, and brain stem regions [12,13]. This neuronal loss leads to a reduction in nerve fibers essential for motor functions [14]. Pathogenesis associated with small nerve fibers in peripheral neuropathy reveals that the accumulation of pathological α-synuclein (phosphorylated) is a primary factor in neuronal death, which may explain the peripheral denervation observed in PD [15,16].

The symptoms usually start slowly and sometimes can drastically worsen over time. Early diagnosis plays a critical role in effective disease management and its delayed progression. The principal diagnosis is clinical, mainly based on motor symptoms. However, in a large part of cases, it is accompanied or preceded by non-motor manifestations, including autonomic dysfunctions [17] such as constipation, sialorrhea or reduced salivary secretion, urinary dysfunction, i.e., urge/incontinence, orthostatic hypotension, excessive sweating, and dysfunctions in thermoregulation. Both motor and non-motor manifestations are generally an expression of the intraneuronal protein accumulation, pathognomonic of the pathology in question. In fact, this phenomenon manifests itself at both the central and peripheral nervous system level and these non-motor symptoms can precede the onset of motor symptoms by many years, as well as constitute a source of disability and reduced quality of life [4,18].

Autonomic dysfunction in PD patients can result in vasoconstriction [19,20,21,22]. In the early stages of PD, this dysfunction disrupts the regulation of body temperature due to excessive vasoconstriction. Autonomic dysfunction is a critical non-motor phenotype of PD. The recent literature suggests that it may serve as a potential biomarker for the early diagnosis of PD [23].

However, an autonomic dysfunction is not always easy to detect; a method could be represented by skin vasomotor response to cold stimuli.

Exposure to cold stimuli triggers a well-characterized physiological response mediated by the sympathetic nervous system, leading to peripheral vasoconstriction in order to minimize heat loss and preserve core temperature. In healthy individuals, this vasoconstrictive response is transient and followed by progressive rewarming once the stimulus is removed. In contrast, patients with autonomic dysfunction—such as those affected by Parkinson’s disease—may exhibit prolonged vasoconstriction or delayed thermal recovery due to impaired sympathetic regulation. The cold stress test (CST) is a widely used method to evaluate these responses, typically involving immersion of the hands or feet in cold water to provoke a cutaneous vasomotor reaction. In this study, immersion of the hands was chosen due to the high density of thermoreceptors and sympathetic innervation in the fingers, as well as their accessibility for thermal imaging. This approach is supported by the previous literature that demonstrated altered skin blood flow and prolonged cooling effects in PD patients using similar CST protocols [23,24].

IRT permits to investigate the involvement of the peripheral autonomic nervous system in PD, which may manifest as an altered cutaneous thermoregulation of the patient’s skin after thermal stress.

It is possible to evaluate alterations in skin thermoregulation and identify variations in localized areas showing abnormal temperature patterns, such as regions of hyperthermia or hypothermia. These specific zones, sometimes referred to as “trigger points” in the context of thermal imaging, are localized regions of the skin that exhibit abnormal thermal behavior in response to stress- or disease-related dysfunction. Identifying such points may help to extrapolate and classify medical data concerning the subject’s physiological status, potentially enhancing the understanding and monitoring of Parkinson’s disease.

The scientific literature is rather sparse regarding studies that examine skin abnormalities in relation to peripheral autonomic dysfunction. One study investigated variations in blood flow regulation at the peripheral limb level in patients with PD [23]. Other research has highlighted discrepancies in skin temperature recovery in parkinsonian patients using thermography [24,25]. Additionally, a further study analyzed distinct cutaneous vasomotor responses to differentiate between early-stage Parkinson’s disease and essential tremor [26]. However, none of the studies conducted to date have assessed whether alterations in peripheral nerve endings are already present in untreated patients at an early stage of the disease. If such alterations were identified, quantifying them could potentially allow their use as prodromal markers.

In this study, a cohort of 16 subjects (8 with PD and 8 healthy controls) underwent monitoring, with infrared images captured of their hands, following a specific protocol established by a preliminary analysis. Experimental results revealed that thermography can detect focal points and regions exhibiting either hyper- or hypothermia across the skin surface and muscular regions. This capability allows for extracting and categorizing precise medical data that are difficult to determine during the early stages of the disease, thus facilitating ongoing monitoring efforts and early diagnosis in Parkinson’s disease research. Moreover, it is possible to extract prodromal biomarkers of the disease and observational framework, allowing timely intervention and management [5,6,9,25,27].

Given the strong link between thermoregulation and autonomic nervous system function, IRT represents a promising tool for assessing autonomic dysfunction in clinical populations. In Parkinson’s disease, where early non-motor symptoms often include impaired autonomic responses, thermographic analysis after a controlled cold stress test may reveal distinctive thermal recovery patterns. While previous studies (e.g., [27,28]) have explored general thermographic alterations in neurological conditions, they did not focus on standardized thermal stimulation or recovery dynamics. In contrast, the present study investigates whether post-CST thermal recovery trends—quantified using TRR and INT parameters—may reveal disease-specific autonomic signatures in PD. The aim of this preliminary observational study is to explore the feasibility of using IRT as a non-invasive tool to detect thermoregulatory differences in PD, and to generate hypotheses for future, larger-scale research.

## 2. Materials and Methods

### 2.1. Measurement Protocol

The measurement protocol hypothesized that the involvement of the peripheral autonomic nervous system in PD patients could be reflected in altered cutaneous thermoregulation. Therefore, it was proposed to assess cutaneous autonomic dysfunction using IRT before and after a thermal stress, i.e., via the cold stress test (CST).

The protocol consisted of the following steps:

- The subject was instructed to sit in a thermo-regulated room of 10 m^2^, after an acclimation of 15 min to an average temperature of 23 °C and a relative humidity of 60%. The ambient temperature was recorded with a digital thermometer at the same time as the infrared measurements.
- The participant was asked to place his arms and hands, spaced 0.5 cm apart, focusing in a frontal view at the front of the camera, on a panel inclined at an angle of 20°, leaving the elbows to rest.
- It was recommended to remove any accessories such as rings or bracelets that could affect the measurement, causing unwanted reflections, with the aim of minimizing the impact of external sources that could lead to errors.
- After a waiting period of about 30 s, to stabilize the temperature of the hands, a first 20 s video was recorded, and the temperature was averaged in order to determine the baseline temperature.
- Then, CST, i.e., the subject was instructed to immerse both hands, up to the wrist, wrapped in latex gloves, in a bowl of cold water at 10 °C. Latex gloves were necessary to avoid subsequent evaporative cooling. Subject was previously recommended to report if it begins to feel pain due to cold water.
- After 2 min of immersion, the gloves were carefully removed from the hands without touching the skin.
- Immediately post-immersion, the skin temperature was continuously monitored from 0 to 10 min.

After the measurement, a thermal imprint remains on the surface for a few minutes, it is therefore necessary to wait about 20 min for its disappearance before proceeding with the next measurement.

**Figure 1:**
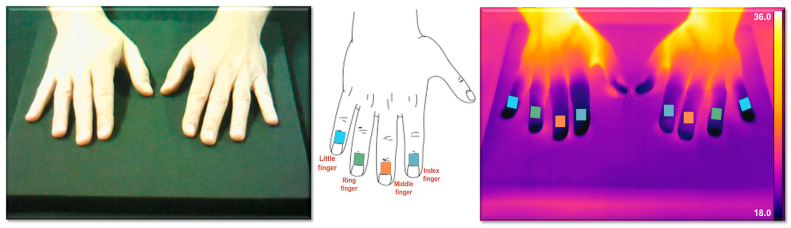
Representation of the regions of interest (ROIs) selected for thermal analysis. The ROIs are located on the distal phalanges of the index, middle, ring, and little fingers of both hands, chosen for their high sensitivity to vasomotor changes and dense sympathetic innervation.

Regions of interest (ROIs) were identified on the distal phalanges of the index, middle, ring, and little fingers of both hands, shown in Figure 1. This anatomical selection is based on prior literature and preliminary measurements, which confirmed the reproducibility and consistency of thermal responses in these areas. The fingers, being highly vascularized and responsive to sympathetic stimuli, provide an optimal site for evaluating cutaneous thermoregulatory dynamics. The figure illustrates the precise positioning of the ROIs used for quantitative analysis of the thermal recovery ratio (TRR) and the integral (INT) parameters.

In addition, preliminary measurements conducted on a small control group (*n* = 4) confirmed the consistency and reproducibility of the thermal response in these areas. These finger regions are known to be highly responsive to peripheral vasoconstriction, making them suitable anatomical targets for cold stress evaluation.

The study was conducted in accordance with the rules of good clinical practice and current legislation and in accordance with the current version of the Declaration of Helsinki. Medical guidelines have been followed [26].

The CST protocol was adapted from previous studies on PD and autonomic dysfunction [18,22], particularly based on the approach described by Shindo et al. [22], who demonstrated the relevance of cold-induced vasoconstriction responses in evaluating autonomic impairment in PD. The use of latex gloves and timing of exposure were selected to reduce evaporative effects and ensure reproducibility.

### 2.2. Participants

The measurements were conducted on 16 different subjects: 8 control subjects (CSs), considered as reference of good health status, and 8 subjects with PD diagnosis between 50 and 70 years of age. CSs were chosen based on the age and sex characteristics of the patients.

The severity of Parkinson’s disease in the PD group was assessed clinically by the referring neurologist. Although subjects were selected to represent mild-to-moderate stages of the disease, standardized clinical staging was not available for all participants.

The exclusion criteria included subjects with diabetes mellitus, using vasoactive medicinal products, with presence of infection or fever before two weeks of testing, a previous history of recent acute myocardial infarction. Furthermore, subjects with no diagnosis of PD with concomitant diagnosis of neuropathies; any other neurological disorders; connective tissue diseases; endocrinopathies, such as thyroidopathies or diseases of the adrenal glands; arterial diseases; and drug abuse that could affect skin temperature and then alter the measurement were excluded.

Each participant was uniquely distinguished for the duration of the study by a three-digit code assigned sequentially from 001. The investigator recorded this code in the case report form, along with the subject name and associated identification number.

### 2.3. Experimental Setup

**Figure 2:**
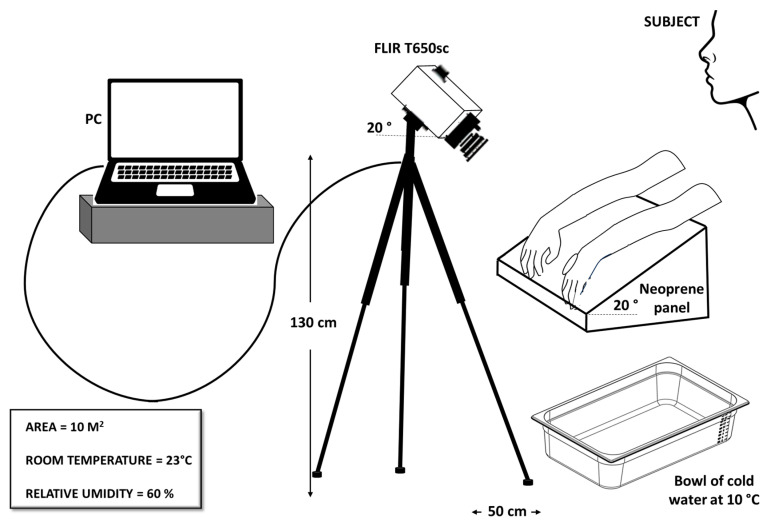
Sketch of the experimental setup.

The experimental setup, shown in Figure 2, consisted of an infrared camera, model T650sc from FLIR Systems, a neoprene contrast panel, and a computer for data processing and storage. A thermal camera is a device designed to detect electromagnetic radiation, which is naturally emitted by the human body, in the infrared spectrum. The T650sc is a high-performance thermal imaging camera meticulously engineered for research and scientific applications. It is equipped with an uncooled vanadium oxide (VOx) microbolometer detector that produces thermal images of 640 × 480 pixels, providing detailed thermal imagery within a spectral range of 7.5 to 14 μm. With a thermal sensitivity of less than 20 mK at 30 °C and a skin emissivity calibration of 0.987, it delivers precise temperature measurements. Its extensive temperature range of −40 to 150 °C enhances its versatility for a broad spectrum of uses, including industrial research, medical diagnostics, and scientific studies. Initially set to an acquisition frequency of 30 Hz for detailed observation of the phenomenon, the frequency was later reduced to 2 Hz to facilitate more manageable data processing.

The camera, inclined of 20°, was mounted on a tripod stand, adjusted to approximately 130 cm above the floor and 50 cm away from the sample. The neoprene panel was employed as a support for the hands, and its opacity was exploited to prevent unwanted reflections. The first problem in a thermographic measurement is to find a uniform surface that can reduce undesirable reflections on which to place the investigating sample. For this purpose, after various tests with different materials, such as paper or wood, the neoprene panel was taken into consideration. It was tilted 20° to promote blood circulation for temperature recovery after CST.

Finally, the acquired IR images were processed using the ResearchIR (Version 4.20) software.

## 3. Results

For brevity, only the results related to the right hands will be reported and finally a study of uncertainly between right and left hands will be presented.

### 3.1. Analyzed Parameters

In order to establish a preliminary parameter as prognostic marker of PD, the thermal recovery ratio (TRR) for each ROI after 10 min was computed as a diagnostic measurement for vasoconstriction in the subjects. The calculation of TRR is depicted in Equation (1) below as ratio of the difference between the temperature 10 min after CST and the temperature immediately afterwards the test (T_0 post CST_) and between the average of the baseline temperature acquired before CST and T_0 post CST_.

**Figure 3:**
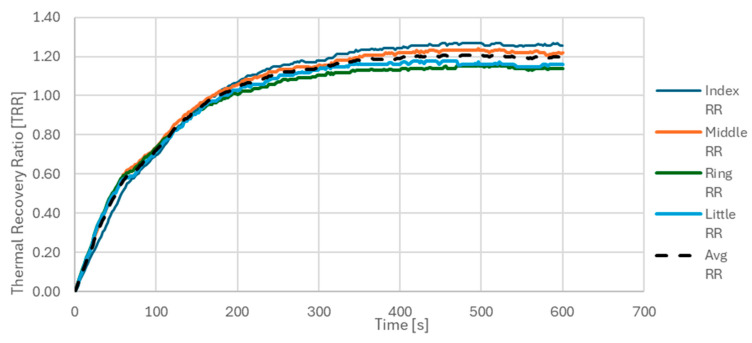
TRR as a function of acquisition time for the four ROIs on the right hand of a control subject. ROIs were defined on the distal phalanges of the index, middle, ring, and little fingers. Colored lines represent TRR values for each individual ROI; the black dashed line indicates the average TRR across all four ROIs. This plot illustrates the typical rewarming trend after cold stress in a healthy subject.

To minimize the error due to the thermal camera measurement in the temperature calculation, only the temperature differences were considered. Furthermore, after calculating the trend of the distribution of the temperature differences for the four individual ROIs, i.e., the TRR for the index, middle, ring, and little fingers, the average of the four TRRs was calculated. Figure 3 shows the trend of the TRR as a function of the acquisition time for the individual ROIs considered. For brevity, the case relating to the right hand of a CS has been reported. The curves relating to the TRRs of the individual ROIs are shown in different colors, while the average of the four TRRs is represented in black with a dashed line.

This parameter was calculated as shown in the equation below, aiming to minimize measurement errors.

(1) $$\mathrm{TRR}=\frac{T_{10_{post\;CST}}-T_{0_{post\;CST}}}{T_{\;baseline\;}-T_{0_{post\;CST}}},$$

Another prognostic parameter considered in this study was the integral of the thermal recovery ratio (INT), defined as the normalized area under the curve (AUC) of the average TRR trend, denoted as f(t), over the 10 min post-CST monitoring period.

**Figure 4:**
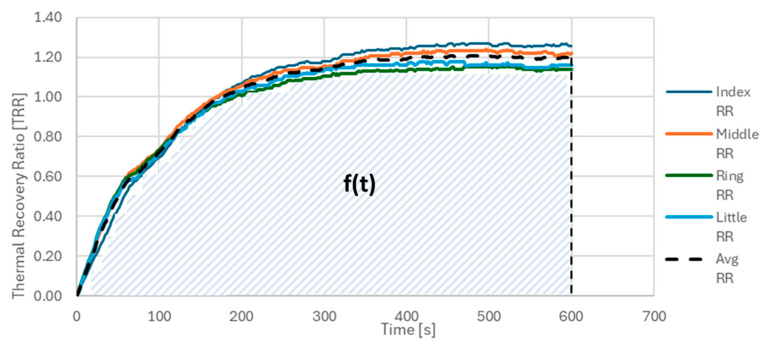
INT parameter calculated as the area covered by the TRR average, divided by the number of points considered.

The INT was calculated as the sum of TRR values over all acquisition points, divided by the total number of acquisition steps, *N* = 600. Each point corresponds to a single frame acquired every second (1 Hz), thus spanning a total of 600 s (10 min). This normalization yields a unitless parameter that reflects the average thermal recovery capacity over time, see Figure 4.

The calculation is expressed as in Equation (2), as follows:

(2) $$\mathrm{INT}=\frac{1}{N}\int f\left(t\right)dt,$$

### 3.2. Thermographic Findings

**Figure 5:**
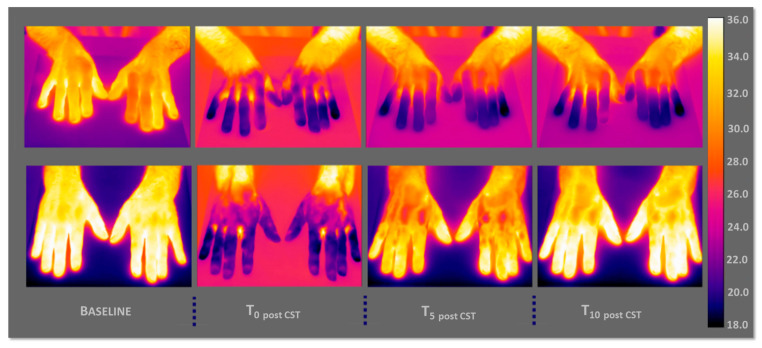
Thermographic images of a PD subject (top) and a control subject (CS, bottom), showing skin temperature distributions at four time points: baseline (left), immediately after CST (T_0 post CST_), after 5 min (T_5 post CST_), and after 10 min (T_10 post CST_). The PD subject shows impaired thermal recovery, while the CS displays a progressive and symmetrical rewarming pattern.

Figure 5 shows, respectively, the thermographic images of a PD (on the top) and a CS (on the below) with a graduated scale of temperature between 18 and 36 °C. More in detail, the basal thermal distribution of the hands before the CST (baseline, reported in the left), immediately afterwards the test (T_0 post CST_), after 5 min (T_5 post CST_), and 10 min of CST (T_10 post CST_).

In the PD case, it is possible to appreciate, by the thermographic view, how the hands show difficulties in thermoregulation, that is, an autonomic dysfunction in the thermal recovery; in fact, they maintain a rather cold temperature.

As regards the case of CS, there is no thermoregulatory problem, and the subject has a normal trend of thermal recovery. The hands are completely heated after 10 min of CST.

**Figure 6:**
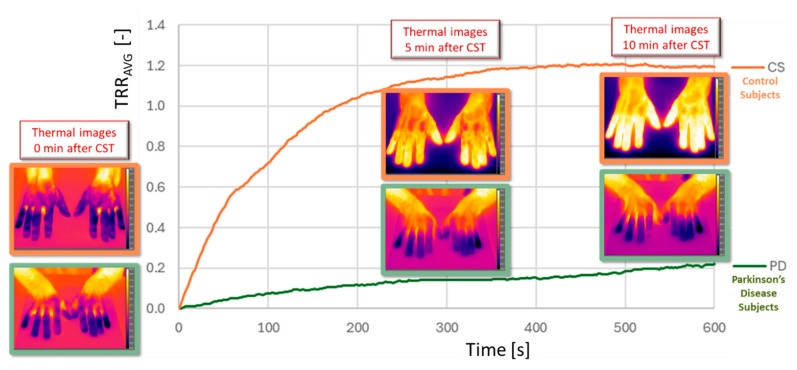
Comparison of average TRR curves for a PD subject (green line) and a CS (orange line), with corresponding thermographic images at 0, 5, and 10 min post-CST. The PD curve remains flat, indicating autonomic dysfunction and delayed rewarming, whereas the CS shows a clear recovery over time.

Figure 6 reports the TRR average as function of the acquisition time calculated in the case of the right hand of a CS (orange line) and a PD (green line) with the corresponding thermal images at 0, 5 min, and 10 min after CST.

The PD case shows a different trend that could be representative of an autonomic dysfunction. Specifically, the TRR maintains an almost constant temperature during the 10 min after CST. Furthermore, while in the case of CS, the max value is 1.2, in the PD case, this value is 10 times less, i.e., 0.2.

**Figure 7:**
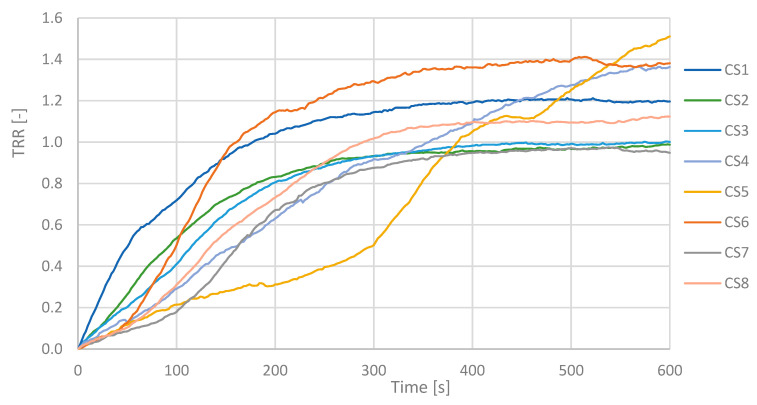
TRR values over time for all control subjects. Each line represents one CS. A consistent rewarming trend is visible across the group, with most TRR values increasing toward 1.0 during the 10 min post-CST period.

**Figure 8:**
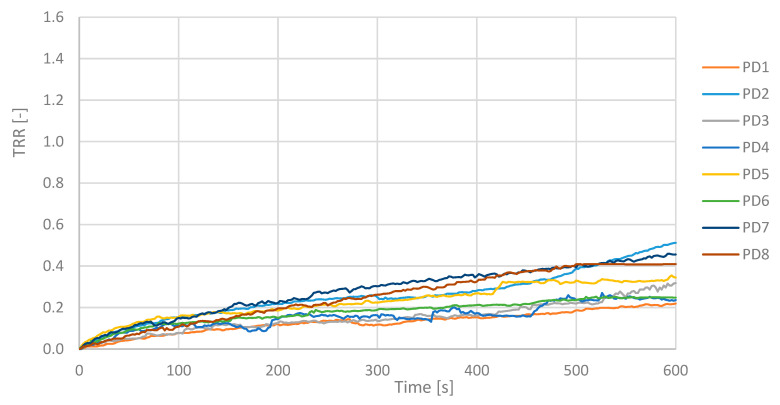
TRR values over time for all Parkinson’s disease subjects. Curves show reduced and delayed recovery compared to controls, with TRR values generally remaining below 0.5 throughout the monitoring period.

In Figure 7 and Figure 8, the TRR values as function of the acquisition time for all subjects are shown for CS (Figure 7) and PD (Figure 8) subjects.

**Figure 9:**
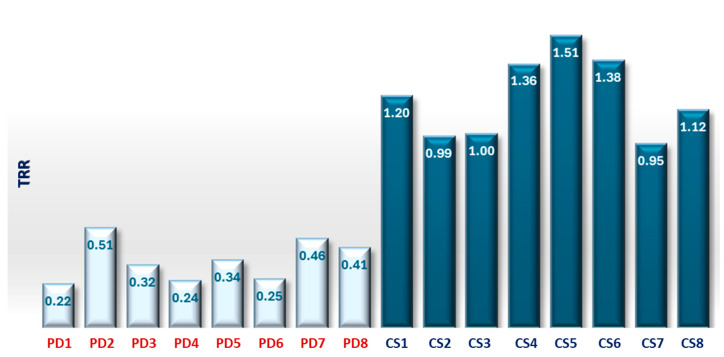
Histogram of mean TRR values for all CS and PD subjects. Control subjects consistently show higher TRR values (around or above 1.0), while PD subjects present significantly lower values, supporting the group-level differences in thermal recovery.

TRR is a parameter that in these measurements showed values less than 0.5 for an illness subject and greater than or next to 1.0 for a healthy subject. PD patients maintain a fairly constant temperature values and show a low TRR. The mean value of the TRR has been calculated for all subjects; PD and CS are reported in the histogram below, see Figure 9.

The TRR for a PD subject shows values between 0.22 and 0.51 according to the severity of the disease, while in a CS, they are next to or greater than 1.00.

**Figure 10:**
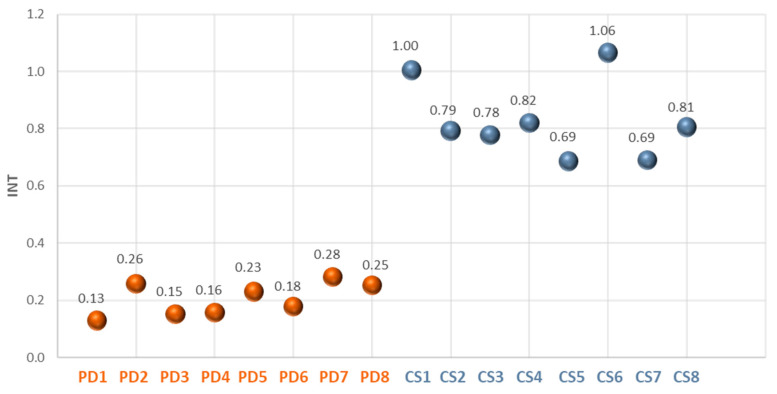
INT values for PD and CS. The INT parameter reflects the area under the average TRR curve over the 10 min observation window. PD subjects exhibit markedly lower INT values, consistent with impaired thermal response. Standard deviation of the results is not reported because lower than 5%.

Similar results also showed the second prognostic parameter, INT, shown in the scatter diagram of Figure 10. PD subjects exhibit lower INT values compared to CS.

The Mann–Whitney U test was applied to compare TRR and INT values between CS and PD groups. While differences did not reach statistical significance (*p* > 0.05), the distribution showed a consistent trend, with CS presenting higher TRR and INT values.

## 4. Discussion

This preliminary observational study aimed to investigate whether infrared thermography (IRT) can reveal alterations in the autonomic thermoregulatory response in subjects with Parkinson’s disease (PD). Compared to healthy subjects, PD patients exhibited significantly lower values in both the thermal recovery ratio (TRR) and the INT parameter. These findings are consistent with reports in the literature suggesting that autonomic dysfunction in PD leads to impaired vasodilation and prolonged vasoconstriction, which can affect thermal recovery after cold stress [19,22,23].

The TRR trends over time demonstrated that control subjects showed a clear and progressive thermal recovery within 10 min, while PD subjects exhibited a flatter curve, indicating slower or absent recovery. The INT parameter, representing the normalized area under the TRR curve, further confirmed this observation, showing markedly reduced values in the PD group. This behavior aligns with previous thermographic studies in PD, which identified slower temperature normalization after cold exposure [24,25,26].

Despite these promising trends, the current findings cannot be generalized due to the limited sample size and the absence of quantitative clinical staging (e.g., UPDRS or Hoehn and Yahr scores). Moreover, although the exclusion criteria ruled out several confounding pathologies, comorbidities known to affect thermoregulation (such as hypertension, depression, or peripheral neuropathies) were not comprehensively documented or controlled. Future studies should include a systematic collection of clinical data, enabling correlation between thermographic findings and disease severity.

Another critical aspect is the specificity of the thermal response. Since no comparison was made with non-PD patients affected by other autonomic disorders, we cannot assert whether the observed thermographic alterations are specific to PD or represent a general autonomic impairment. The inclusion of disease controls (e.g., subjects with diabetic neuropathy or multiple system atrophy) would help clarify this issue. From a methodological perspective, IRT proved to be a non-invasive and sensitive tool, capable of capturing subtle changes in skin perfusion dynamics. However, technical limitations remain, such as sensitivity to ambient conditions, the need for strict measurement protocols, and the challenges of standardizing ROI selection and temperature thresholds.

In summary, this study provides preliminary evidence supporting the potential of IRT to detect altered thermoregulatory patterns in PD. These findings open the way to future investigations using larger cohorts, detailed clinical characterization, and comparative analyses with other neurological and autonomic conditions. While diagnostic applications are premature at this stage, thermography may serve as a supportive monitoring tool or a component in multimodal diagnostic frameworks.

Several limitations must be acknowledged in this study. First, the sample size of only 16 subjects (8 PD, 8 control) is relatively small and does not allow for strong statistical inference or generalization. Second, standardized quantitative clinical assessments of Parkinson’s disease severity, such as the Unified Parkinson’s Disease Rating Scale (UPDRS) or Hoehn and Yahr staging, were not available for all participants, preventing detailed correlation between disease severity and thermoregulatory alterations. Third, although some exclusion criteria were applied (e.g., diabetes, vasoactive medication use, recent infections, other neurological or systemic diseases), other influencing factors were not systematically controlled or assessed. Fourth, individual variability in thermoregulatory response, influenced by factors such as age, sex, skin characteristics, and undiagnosed or subclinical comorbidities, may have contributed to the observed differences and should be carefully considered. Fifth, while trends in TRR and INT parameters indicated group-level differences between PD and control subjects, these did not reach statistical significance, likely due to the small sample and exploratory nature of the study. Finally, no diagnostic thresholds for TRR or INT can currently be established; the findings should be considered as preliminary observations intended to guide future investigations.

These limitations underline the preliminary nature of the findings and the need for larger-scale, clinically stratified investigations to confirm and expand upon these observations. Although this study did not include direct comparisons with established diagnostic tools for autonomic dysfunction, it is important to situate IRT within the broader clinical context. Gold-standard assessments in Parkinson’s disease typically involve cardiovascular autonomic function testing, such as heart rate variability (HRV), tilt-table testing, and the Valsalva maneuver, as well as clinical scales like SCOPA-AUT and COMPASS-31. These methods provide well-validated quantitative measures of autonomic impairment. Infrared thermography, in contrast, offers a non-contact, imaging-based approach that can reveal peripheral vascular responses potentially linked to autonomic dysfunction. While IRT does not replace established autonomic testing, it may serve as a valuable adjunct, especially in early-stage patients or in settings where conventional methods are not available or tolerated.

Future studies should include parallel assessments with clinical scales and physiological measurements to better define the role of IRT in the diagnostic pathway. Ultimately, with a substantial number of measurements, it will be possible to develop a mathematical model that better approximates the calculated temperature distribution function, as has been done previously in other fields [29].

## 5. Conclusions

This study explored the use of infrared thermography (IRT) to assess thermoregulatory response following a cold-stress test in subjects with Parkinson’s disease (PD) compared to healthy controls. The results showed consistent differences in thermal recovery trends, with PD subjects exhibiting reduced rewarming capacity, likely associated with autonomic dysfunction. The proposed parameters, TRR and INT, revealed potential group-level distinctions between PD and control subjects.

However, the small sample size, the absence of quantitative clinical staging, and the lack of detailed data on comorbidities or disease controls significantly limit the generalizability and clinical applicability of these findings.

The present study must therefore be considered observational and exploratory. No diagnostic thresholds can currently be proposed. The findings should be interpreted as preliminary indications of thermographic trends in PD, serving as a foundation for future investigations. Larger studies with broader clinical characterization are needed to assess the sensitivity, specificity, and potential role of IRT in PD monitoring or diagnosis.

In addition to the limited sample size, individual variability in cold response and skin thermoregulation, potentially influenced by demographic or physiological factors, further constrains the scope of interpretation. Methodological factors, including sensitivity to ambient conditions, the need for strict measurement protocols, and the challenges of standardizing ROI selection and temperature thresholds, must also be considered. Furthermore, no comparisons were made with subjects affected by other autonomic or neurological disorders, limiting conclusions on the specificity of IRT findings for PD. Similarly, direct comparisons with established diagnostic tools, such as heart rate variability (HRV), tilt-table testing, and clinical scales like SCOPA-AUT or COMPASS-31, were not performed, highlighting the need for parallel assessments in future studies.

This study has several limitations, the most significant being the small sample size and the absence of standardized clinical staging and detailed comorbidity assessment. These factors limit the generalizability of the findings and restrict the ability to draw definitive conclusions. Future work should include larger, stratified cohorts and consider the integration of IRT with conventional autonomic testing tools. Additionally, alternative CST methodologies, such as cold immersion of the foot with thermal monitoring of the dorsal hands, could be explored to assess the robustness and flexibility of the thermographic approach under different stress conditions.

Future research will aim to expand the sample size and include longitudinal assessments, as well as correlations with established clinical biomarkers and autonomic function tests, to validate and refine the observed thermographic patterns.

## Data Availability

Data will be made available upon reasonable request to the corresponding author.
